# What Are the Human-Specific Aspects of Neocortex Development?

**DOI:** 10.3389/fnins.2022.878950

**Published:** 2022-04-14

**Authors:** Felipe Mora-Bermúdez, Wieland B. Huttner

**Affiliations:** Max Planck Institute of Molecular Cell Biology and Genetics, Dresden, Germany

**Keywords:** human-specific, neurogenesis, neocortex, stem cells, neuroepithelial cells, radial glia, chimpanzees, Neandertals

## Abstract

When considering what makes us human, the development of the neocortex, the seat of our higher cognitive abilities, is of central importance. Throughout this complex developmental process, neocortical stem and progenitor cells (NSPCs) exert a priming role in determining neocortical tissue fate, through a series of cellular and molecular events. In this Perspective article, we address five questions of relevance for potentially human-specific aspects of NSPCs, (i) Are there human-specific NSPC subtypes? (ii) What is the functional significance of the known temporal differences in NSPC dynamics between human and other great apes? (iii) Are there functional interactions between the human-specific genes preferentially expressed in NSPCs? (iv) Do humans amplify certain metabolic pathways for NSPC proliferation? and finally (v) Have differences evolved during human evolution, notably between modern humans and Neandertals, that affect the performance of key genes operating in NSPCs? We discuss potential implications inherent to these questions, and suggest experimental approaches on how to answer them, hoping to provide incentives to further understand key issues of human cortical development.

## Introduction

In this Perspective article, we discuss several key questions in the field of *neurogenesis in the developing neocortex*. In light of our own research experience, we focus on neocortical stem and progenitor cells (NSPCs) and discuss noteworthy human-specific aspects pertaining to these cells. We include both, (i) human-specific aspects that are qualitative in nature, such as the appearance of human-specific genes during evolution that induce NSPC proliferation; and (ii) human-specific aspects that are quantitative in nature, such as temporal differences in NSPC dynamics between humans and other hominids, i.e., non-human great apes. We define “human-specific” aspects as those found in present-day (”modern”) humans as well as in archaic, extinct humans such as Neandertals and Denisovans. In contrast, we define “modern human-specific” aspects as those found only in present-day humans, that is, features that arose after the divergence of the ancestors of modern humans from those of Neandertals and Denisovans. In discussing human-specific and modern human-specific aspects of NSPCs, we highlight arising questions and suggest approaches on how to answer them.

## Neocortical Stem and Progenitor Cell Types—Are There Human-Specific Subtypes?

**FIGURE 1:**
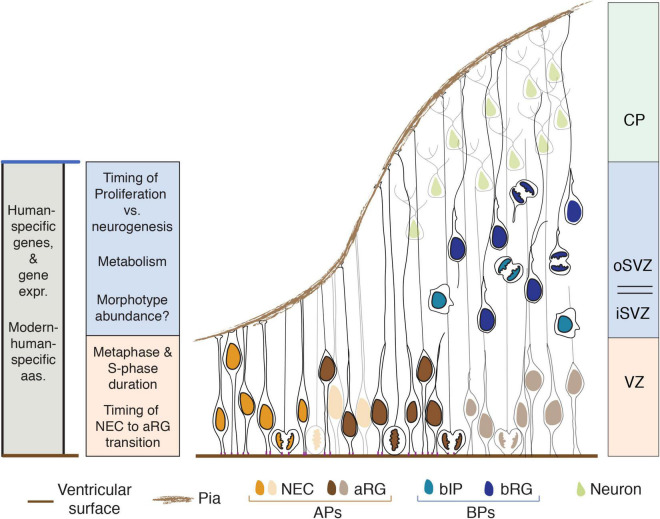
Human-specific aspects of NSPCs that influence neocortex development. Aspects listed on the left are color-coded according to the germinal zone(s) where they play a role (right side) or shown in gray if they apply to all germinal zones (expr., expression; aas., amino acid residues). The main cell types per zone are shown; VZ, ventricular zone; subventricular zone (SVZ), subdivided in inner (iSVZ) and outer (oSVZ) SVZ; CP, cortical plate; NEC, neuroepithelial cells; aRG, apical radial glia; APs, apical progenitors; bIP, basal intermediate progenitor; bRG, basal radial glia; BPs, basal progenitors. To illustrate the progression from NECs to aRG to BPs to neurons (from left to right), the nuclei of NECs and aRG are first shown in full color and then in a pale version of the respective color.

The primary criterion for the classification of the various types of NSPCs in the developing neocortex is the germinal zone where the cell body of a given NSPC type resides, and hence where it undergoes mitosis (Taverna et al., 2014). This is tightly linked to a fundamental cell biological feature of the developing cortical wall, its apical-basal polarity. Thus, the various NSPC types residing in the primary germinal zone that is located at the apical side of the cortical wall, the ventricular zone (VZ), are collectively referred to as apical progenitors (APs). APs include (i) neuroepithelial cells (NECs), (ii) apical radial glia (aRG, also referred to as ventricular radial glia), and (iii) apical intermediate progenitors (aIPs). All of these NSPC types undergo mitosis at the ventricular, i.e., apical, surface of the cortical wall, which follows from the fact that the centrosomes needed to form the mitotic spindle are tethered to the apical cell cortex during the entire interphase of these NSPC types [Figure 1; AP types and the VZ are reviewed in detail in Bystron et al. (2008), Taverna et al. (2014), Mora-Bermúdez et al. (2016b)].

The various NSPC types residing in the secondary germinal zone, the subventricular zone (SVZ), are collectively referred to as basal progenitors (BPs). This term reflects the fact that, with regard to the above-mentioned apical-basal polarity axis of the developing cortical wall, the cell bodies of BPs are located basally to those of APs. BPs include (i) basal radial glia (bRG, also referred to as outer radial glia), and (ii) basal intermediate progenitors (bIPs). In contrast to APs, none of the BPs contact the ventricle. Their centrosomes—like their cell bodies—are not tethered to the apical side but are instead located in the SVZ, and hence undergo mitosis there [Figure 1; BP types and the SVZ and its subdivisions are reviewed in detail in Bystron et al. (2008), Taverna et al. (2014), Dehay et al. (2015)].

A secondary criterion to distinguish between the various types of APs and BPs is their morphology, specifically the presence, number, orientation and structure of cell extensions, i.e., cell processes. Thus, among the APs, both NECs and the canonical aRG extend a radial process that contacts the basal lamina; in the case of the aRG, this basal process passes through the SVZ, intermediate zone and cortical plate and exhibits a distinct molecular composition. In contrast, the so-called truncated aRG and the aIPs extend a radial process in the basal direction that does not contact the basal lamina. These radial processes of the various AP types persist throughout their cell cycle, including mitosis (Figure 1; Florio and Huttner, 2014; Taverna et al., 2014; Kalebic and Huttner, 2020).

Among the BPs, bRG characteristically extend a basal process (which may or may not reach the basal lamina) and/or an apically directed process; both types of processes are typically also observed at mitosis. In contrast, bIPs lack such radial processes but are multipolar in interphase, i.e., extend short processes in various directions, and retract these processes during mitosis (Figure 1; Taverna et al., 2014; Kalebic and Huttner, 2020).

An additional criterion to distinguish between the various types of APs and BPs is the nature of their progeny. Among the APs, mitotic NECs and aRG give rise to other NSPCs (i.e., APs and/or BPs), whereas aIPs give rise to neurons. Similarly, among the BPs, diverse functional subtypes of bRG and bIPs can be distinguished, depending on whether their progeny consists of BPs, neurons, or both (Figure 1; Betizeau et al., 2013; Taverna et al., 2014).

Application of these criteria, when comparing the NSPCs of various mammals, has revealed major changes in the relative abundance of the various NSPC types that also apply to human evolution. First, when compared to the embryonic neocortex of the mouse, a canonical lissencephalic model system, the fetal neocortex of the gyrencephalic human exhibits a dramatic increase in the ratio of BPs over APs. Second, there is a huge increase in the ratio of bRG over bIPs (Florio and Huttner, 2014; Taverna et al., 2014; Borrell, 2019). However, neither of these increases are human-specific, as they are also observed when comparing the embryonic mouse neocortex to that of the macaque, a non-human primate (Betizeau et al., 2013). Yet, a more detailed analysis of the morphology of BPs revealed an increased relative abundance of the two bRG subtypes with a bifurcated basal process in fetal human neocortex compared to the embryonic neocortex of the ferret, a gyrencephalic carnivore (Kalebic et al., 2019). Moreover, these two bRG subtypes have not been reported for the fetal macaque neocortex (Betizeau et al., 2013), raising the possibility that the increased relative abundance of these two bRG subtypes could be a hominid or human-specific feature.

From these data, the key challenge emerges to compare the cellular and molecular features of human NSPCs with those of our closest non-extinct relatives, the non-human great apes (Mora-Bermúdez et al., 2021). A huge advance toward this goal has been the development of the brain organoid technology (Kadoshima et al., 2013; Lancaster et al., 2013; Pasca, 2018; Chiaradia and Lancaster, 2020; Velasco et al., 2020). However, although brain organoids are extremely useful models, it must be born in mind that they do not fully recapitulate all aspects of cortical development, especially those related to the SVZ and neuronal layers (Heide et al., 2018). Nonetheless, the first study focusing on the comparison of NSPCs in human, chimpanzee and orang-utan did identify a difference in the mitosis of APs between human on the one hand, and chimpanzee and orang-utan on the other—the prolongation of metaphase by 50% in human (Mora-Bermúdez et al., 2016a). While this metaphase prolongation is a human-specific, and potentially a modern human-specific, feature of APs, we do not think that it reflects the existence of a truly human-specific, or even modern human-specific, AP subtype, as discussed in the following section.

How, then, should a search for the potential existence of human-specific NSPC subtypes be conducted? We find that a molecular and a cellular approach, alone or in combination, are most promising. As to a molecular approach, single-cell transcriptomics as performed in the above-mentioned study (Mora-Bermúdez et al., 2016a) and expanded in subsequent studies toward a systems level (Kanton et al., 2019; Pollen et al., 2019) have great potential as they have revealed a number of interesting genes with expression differences between human and non-human NSPCs and neurons, such as integrin beta 8 (*ITGB8*) and insulin receptor (*INSR*). The key challenge here will be to determine whether or not such gene expression differences translate into unique, human-specific physiological features of the NSPCs concerned and, if so, whether they could suffice to justify the existence of potential human-specific NSPC subtypes.

As to a cellular approach, in addition to light microscopy techniques, the advent of the serial block face scanning electron microscopy (SBF-SEM) technology (Wanner et al., 2015) would allow an analysis of the subcellular structure of human vs. chimpanzee NSPCs in cortical tissue at unprecedented detail and resolution. Of note, the identity of cells in tissue samples for SBF-SEM can be correlated with light microscopic data by first imaging of fluorescently labeled cells in a light-microscopic 3D volume, followed by EM embedding and 3D imaging with SBF-SEM. Importantly, the SBF-SEM approach would be applicable not only to brain organoids but, as it starts with fixed tissue, also to authentic neocortical tissue, which can be obtained from fetal samples not only of human but also—albeit extremely rarely—of chimpanzee. Should this approach lead to the identification of differences in the subcellular structure of a given NSPC type between human and chimpanzee, the challenge then will again be to determine whether or not such differences underlie a unique, human-specific feature of the NSPC type concerned such that it can be regarded as a human-specific subtype. This could be explored using human vs. chimpanzee brain organoids.

In sum, while the evidence obtained so far from organoid studies suggests that the principal types of NSPCs are conserved between humans and non-human great apes, the search for potential human-specific, or even modern human-specific, NSPC subtypes remains a crucial challenge. Despite the limitations of brain organoids, it appears worthwhile to concentrate future research efforts on comparative studies of the less-explored SVZ of human vs. non-human great apes. Such studies should focus on morphological differences as well as the potential impact of human-specific genes and/or differences in the expression patterns of genes common to all hominids.

## Temporal Differences in Neocortical Stem and Progenitor Cell Dynamics Between Human and Non-Human Great Apes—What Is Their Functional Significance?

### Metaphase Duration of Mitotic Apical Progenitors

As mentioned above, we previously reported a prolongation by 50% of the metaphase during the mitosis of human organoid APs compared to chimpanzee and orang-utan (Mora-Bermúdez et al., 2016a). This human-specific prolongation is intriguing because it was not observed in non-neural cells, and also because the known role of metaphase in neurogenesis has, so far, been limited to setting up the position and orientation of the mitotic spindle, and hence of the division plane, for symmetric vs. asymmetric divisions of APs (Homem et al., 2015; Mora-Bermúdez and Huttner, 2015, 2018). Since the metaphase prolongation of human APs was not accompanied by cleavage plane orientation differences, this suggested a previously unknown role in neurogenesis and cortical evolution. Moreover, this human-specific AP metaphase prolongation arose during the neural differentiation of organoids and occurred only during the early phase of neurogenesis, suggesting that it was linked to the expansion of APs that characteristically takes place during this phase (Mora-Bermúdez et al., 2016a).

Given that metaphase is the last phase of mitosis where chromosomes prepare for segregation, a longer metaphase may have a role in ensuring that all chromosomes are ready for accurate segregation (Foley and Kapoor, 2013; Musacchio, 2015). This prolongation could result from a more “stringent” threshold by the spindle assembly checkpoint, which may require more time to be satisfied and signal for anaphase entry. A potential benefit of metaphase prolongation could be a minimization of chromosome segregation errors, such as lagging chromosomes that may be distributed to the wrong daughter cell, may form micronuclei, or may become damaged or lost during cytokinesis and abscission. In the case of human APs undergoing expansion, a minimization of chromosome segregation errors due to metaphase prolongation would have consequences for the entire progeny of APs, that is, BPs as well as cortical neurons and glial cells. Recent data has brought strong support to these concepts (Mora-Bermúdez et al., 2022).

In this context, it is interesting to note that the metaphase time required for the preparation for chromosome segregation does not appear to be correlated to the number of chromosomes, as both chimpanzees and orangutans have 2N = 48 chromosomes, i.e., 2 *more* than humans, yet experience a shorter AP metaphase. Also, despite mice having just 4 chromosomes (≈9%) less than humans (2N = 42), mouse APs have a metaphase that is around 60% shorter (Mora-Bermúdez et al., 2016a).

### Developmental Timing of the Transition Between Neocortical Stem and Progenitor Cell Types

A recent organoid study has provided morphological and molecular evidence that the transition from NECs to aRG is delayed in human compared to gorilla. On the morphological side, early organoid human NSPCs remained more time with a shorter basal process and a larger apical contact than those of gorilla, better matching the canonical NEC structure. On the molecular side, the transcription factor ZEB2, identified as a driver of the NEC-to-aRG transition, was correspondingly expressed later in human than gorilla NSPCs (Benito-Kwiecinski et al., 2021). This developmental delay would allow for a longer expansion phase of the highly proliferative NECs (Götz and Huttner, 2005), potentially increasing the resulting pool of aRG, from which all subsequent neocortical neural cells are derived.

A second example of a developmental delay in the transition of human vs. non-human ape NSPCs was found when comparing proliferation and neurogenesis in cerebral organoids of humans and chimpanzees. Specifically, the transition from proliferating NSPCs to neurogenic NSPCs occurred more slowly in human than chimpanzee. Consistent with this observation, single-cell mRNA sequencing suggested a higher proliferative potential of human than chimpanzee APs. Also, human APs showed a longer S-phase than those of chimpanzees (Mora-Bermúdez et al., 2016a), and a longer S-phase has previously been correlated with a higher proliferative potential of APs in mice and macaque (Kornack and Rakic, 1998; Arai et al., 2011; Dehay et al., 2015), suggesting that this may also be the case in hominids.

Together, these studies suggest that, while no evidence exists so far for the existence of human-specific progenitors, the proliferative phases of NSPCs are prolonged in a human-specific fashion by delaying the onset of the neurogenic phases. This allows for a greater expansion of the NSPC pool, and of the subsequent neuronal progeny. This is consistent with the concept that the timing of developmental transitions, and the consequent total length of the neurogenic period, play major roles in determining the number of neurons produced by NSPCs and in the growth of the neocortex (Rakic, 1995, 2000; Lewitus et al., 2014; Stepien et al., 2020; Mora-Bermúdez et al., 2021). Further elucidating the cellular and molecular underpinnings of controlling the lengths of the proliferation phases and of the neurogenic period is likely to be very interesting.

## Human-Specific Genes Preferentially Expressed in Neocortical Stem and Progenitor Cells—Are There Functional Interactions?

A major issue of relevance for potential human-specific aspects of NSPC biology pertains to human-specific genes that are preferentially expressed in these cells (Heide and Huttner, 2021). A previous search identified 15 such genes (Florio et al., 2018). Two of these, *ARHGAP11B* and *NOTCH2NL*, have already been under intense investigation, but little is known about the role of the other 13 genes. Both *ARHGAP11B* and *NOTCH2NL* have been shown to promote NSPC proliferation and to increase NSPC levels. Specifically, *ARHGAP11B* increases the proliferation and level of BPs, notably of bRG (Florio et al., 2015, 2016; Kalebic et al., 2018; Heide et al., 2020). *NOTCH2NL-A*, one of the three human-specific *NOTCH2NL* genes, also increases the proliferation and level of BPs, however with a preference for bIPs rather than bRG (Florio et al., 2018). In contrast, *NOTCH2NL-B* has been reported to increase AP levels (Suzuki et al., 2018). Interestingly, the mechanisms of action of the ARHGAP11B and NOTCH2NL proteins are distinct. ARHGAP11B is localized in mitochondria, where it induces a specific metabolic pathway, that is, glutaminolysis (Namba et al., 2020), a hallmark of highly proliferative cells (see below). The NOTCH2NL proteins, however, have been found to enhance the activation of the Notch receptor and hence appear to exert their effects at the plasma membrane and/or the cell cortex (Fiddes et al., 2018; Suzuki et al., 2018). Despite this different subcellular localization, investigating whether there is a functional synergism of ARHGAP11B and NOTCH2NL with regard to NSPC proliferation is an important question.

Furthermore, possible functional interactions between ARHGAP11B and/or NOTCH2NL with the proteins encoded by the other 13 human-specific genes preferentially expressed in NSPCs is a key issue for future investigations. In this context, it is intriguing to note that the three *NOTCH2NL* genes co-evolved with human-specific members of the *NBPF* (Neuroblastoma breakpoint family) gene family (Popesco et al., 2006; Fiddes et al., 2019). Specifically, NOTCH2NL-A co-evolved with NBPF10, NOTCH2NL-B with NBPF14, and NOTCH2NL-C with NBPF19. The NBPF genes and proteins are interesting for various reasons. First, they increase in number in the human (Vandepoele et al., 2005). Second, concomitant with this increase, there is a striking increase in the number of the so-called Olduvai domains, previously known as DUF1220 (domain of unknown function 1220) (Popesco et al., 2006), and a high number of copies of this domain has been found to be correlated with brain size (O’Bleness et al., 2012). Hence, in line with previous considerations (Heide and Huttner, 2021), these findings suggest that exploring a potential role in NSPC proliferation of those *NBPF* genes that are human-specific, as well as possible functional interaction with the *NOTCH2NL* genes, appears to be a very worthwhile future effort.

## Neocortical Stem and Progenitor Cell Metabolism—Do Humans Amplify Certain Pathways?

As mentioned in the previous section, the human-specific gene *ARHGAP11B* promotes BP proliferation *via* stimulation of glutaminolysis (Namba et al., 2020). This metabolic pathway is also involved in the stimulation of NSPC proliferation by Mcph1 (Journiac et al., 2020). Although the *MCPH1* gene is not human-specific, this study has provided a link between primary microcephaly and glutaminolysis. In this metabolic pathway, glutamine is converted to glutamate which is then converted to α-ketoglutarate (αKG), an intermediate in the TCA cycle (also called citric acid or Krebs cycle). In a recently proposed concept, referred to as the “three-quarter TCA cycle concept” (Namba et al., 2021), it has been hypothesized that the increase in αKG levels due to the ARHGAP11B-induced stimulation of glutaminolysis would result in an increased level of oxaloacetate, the final intermediate in the TCA cycle; oxaloacetate could then serve as a source for various anabolic processes that in turn would promote cell cycle progression and hence BP proliferation. The use of oxaloacetate as a source for anabolic processes rather than as a “starter” of the TCA cycle by the aldol condensation reaction with acetyl-CoA would be particularly relevant under conditions when acetyl-CoA is used for other purposes such as fatty acid synthesis, or when its availability for citric acid synthesis is reduced. The latter is the case when glycolysis proceeds all the way to lactate, rather than ending with pyruvate. Remarkably, glycolysis proceeding all the way to lactate has been shown to be beneficial for NSPC proliferation. In other words, an increase in the proliferative capacity of BPs, which is thought to be crucial for the expansion of the neocortex during human evolution, seems to involve the synergism of two metabolic pathways, glycolysis ending at lactate (rather than pyruvate), and a three-quarter TCA cycle starting at αKG and ending at oxaloacetate (Namba et al., 2021).

This insight raises the questions of (i) whether the increase in the proliferative capacity of BPs in the course of the evolutionary expansion of the human neocortex involves yet additional metabolic pathways, besides glycolysis ending at lactate and glutaminolysis, and whether these may operate synergistically with the former two; and (ii) whether humans amplify the activity of any of these pathways compared to chimpanzees. The findings that *ARHGAP11B* is a human-specific gene that achieves its effect on BP proliferation *via* stimulation of glutaminolysis (Namba et al., 2020) suggests that, at least for this metabolic pathway, the second scenario applies. Future studies comparing the metabolism of NSPCs in human vs. chimpanzee cerebral organoids are likely to be a promising approach to obtain further insight into this fascinating topic.

## Evolutionary Aspects—Are There Differences in Key Players of Neocortical Stem and Progenitor Cell Dynamics Between Modern Humans vs. Neandertals?

The human-specific NSPC features discussed so far have probably been shared not only among present-day humans, but also with other closely related archaic hominins, because the genomic underpinnings of those features have also been found in extinct archaic humans, such as Neandertals and Denisovans. These genomic underpinnings, while being important stepping stones, are, however, insufficient to arrive at the full genomic and physiological constitution of present-day humans as compared to extinct archaic humans.

Ancient DNA sequencing and bioinformatics studies have uncovered catalogues of differences between archaic and modern humans (Meyer et al., 2012; Prüfer et al., 2014). Both coding and non-codding differences have been found, with little less than 100 single amino acid differences in almost as many proteins, and around 30,000 non-coding nucleotide differences, fixed or nearly fixed, throughout the genome of modern humans (Meyer et al., 2012; Prüfer et al., 2014; Kuhlwilm and Boeckx, 2019).

Recent efforts to study the physiological relevance of such differences for brain development have focused on the potential physiological consequences of single amino acid differences. For example, human organoids with the ancestral amino acid variant of splicing regulator NOVA1 (isoleucine instead of valine) were reported to be smaller and have more cell death, to have a more uneven external surface, and to have gene expression differences in synaptic and glutamatergic markers compared to those with the modern human variant (Trujillo et al., 2021). However, it has been questioned whether these phenotypes were really due to the single amino acid substitution in NOVA1 (Herai et al., 2021; Maricic et al., 2021).

An intriguing question that arises is whether such single amino acid differences act indeed in isolated fashion, or if they may potentially act together with others in complex cellular structures and pathways. It is interesting to note that many of these differences can be classified by gene ontology as having functions in common pathways. One example of several amino acid differences between archaic and modern humans acting in the same process could be those found in proteins with functions in the microtubule cytoskeleton and the mitotic spindle (Pääbo, 2014; Prüfer et al., 2014). More specifically, three proteins that collectively contain six single amino acid differences, namely KIF18a (or Kinesin 8) with one amino acid difference, KNL1 (or CASC5) with two differences, and SPAG5 (or Astrin) with three differences, have been shown to be involved in kinetochore function and chromosome segregation during cell division (Manning et al., 2010; Hafner et al., 2014; Zhang et al., 2017).

It is intriguing that such a relatively high number of amino acid substitutions is associated with one cellular structure. In this context, two key functions of the kinetochore are the anchoring of chromosomes to kinetochore microtubules for segregation to the daughter cells, and being a crucial site for the assembly and signaling of the spindle assembly checkpoint (Musacchio and Salmon, 2007). Interestingly, both of these processes occur during prometaphase and metaphase. Also, the six amino acid variants found in Neandertals are identical to those found in chimpanzees and other non-human great apes. Recent data has shown that the functions of these coding differences between modern and archaic humans are related to the metaphase prolongation observed in modern human vs. chimpanzee and orang-utan organoid APs (Mora-Bermúdez et al., 2016a, 2022).

Further studies that convert all modern human-specific amino acid variants potentially involved in one process to the ancestral versions (i.e., an “ancentralization,” or “neandertalization”) will therefore be key to explore potential common functions. Studies in appropriate model systems that also perform the converse changes, from the ancestral to the modern human variant (i.e., a “modern-humanization,” or “sapienization”) will be equally important to corroborate and obtain a more complete picture, especially since potential phenotypes of extinct species cannot be tested in native tissue.

## Conclusion

The five questions addressed in this Perspective article concern features of NSPCs which, from our research experience, seem to be of particular relevance to gain further insight into human-specific, and even modern human-specific, aspects of neurogenesis in the developing neocortex. Thus, while the choice of these five questions likely reflects personal bias, we hope that the potential implications inherent to these questions, and the experimental approaches suggested to answer them, will serve as incentives for future efforts to understand key issues of human cortical development. The focus on NSPCs presented here is surely just one facet of this complex developmental process. Thus, topics not addressed in this Perspective article, such as neuronal migration and maturation, synaptogenesis, neuronal circuit formation, gliogenesis, developmental plasticity, and interactions with the environment, are clearly equally essential for trying to obtain a reasonably complete picture of the series of developmental events that contribute in a crucial way to what makes us human.

## Author Contributions

Both authors contributed equally to writing and revising the manuscript and approved the submitted version.

## Conflict of Interest

The authors declare that the research was conducted in the absence of any commercial or financial relationships that could be construed as a potential conflict of interest.

## Publisher’s Note

All claims expressed in this article are solely those of the authors and do not necessarily represent those of their affiliated organizations, or those of the publisher, the editors and the reviewers. Any product that may be evaluated in this article, or claim that may be made by its manufacturer, is not guaranteed or endorsed by the publisher.
